# Buffalo Medical College

**Published:** 1849-05

**Authors:** 


### EDITORIAL DEPARTMENT.

*Buffalo Medical College. Commencement.*—The public commencement exercises at this Institution, took place on the evening of the 18th ult., at the First Presbyterian Church. The exercises were opened with prayer by the Rev. Mr. Thomson, pastor of the church. Music by the choir of the church followed. The degrees were then conferred by Dr. Thomas M. Foot, in the absence, from the city, of the Hon. Millard Fillmore, Chancellor of the University, upon the following gentlemen:—

John D. Hill, N. Y.; Geo. W. Smith, N. Y.; John W. Jones, Ohio; Chas. H. Swain, N. Y.; Dix. A. Shevalier, N. Y.; Eldred P. Grey, N. Y.; N. R. Derby, Pa.; F. F. Hoyer, N. Y.; W. D. O. K. Strong, N. Y.; Levi Aldrich, Michigan; Joel Green, jr., Ohio; Wm. H. Watkins, N. Y.; Nelson Saunders, N. Y.; Judson Bowen, N. Y.; W. D. Fitch, Canada West; Daniel Wilson, Canada West; Wm. Henry Borden, N. Y.; Robert Mitchell, jr. N. Y.; Robert Gilfillan, Michigan.—Total 19.

After the degrees were conferred, a valedictory address to the graduates was pronounced by Prof. C. A. Lee, replete with elevated sentiments, and valuable counsel, conveyed in a chaste and classical style. The delivery of the address occupied an hour and a quarter, and we have rarely known the attention of a mixed auditory so perfectly preserved as it was on this occasion.

The exercises closed with music by the choir, and benediction by the Rev. Mr. Thomson.

